# Coverage of large soft tissue defects of the lower limb and foot with superficial inferior epigastric artery flap

**DOI:** 10.3389/fsurg.2024.1424681

**Published:** 2024-10-24

**Authors:** Dong Liu, Xingwen Xie, Ping An Chu, Xin Zhou, Lin Luo, Ning Li

**Affiliations:** ^1^ School of Clinical Chinese Medicine, Gansu University of Chinese Medicine, Lanzhou, Gansu, China; ^2^ Department of Orthopaedics, The Affiliated Hospital of Gansu University of Chinese Medicine, Lanzhou, Gansu, China; ^3^ Department of Orthopaedics, The Affiliated Traditional Chinese Medicine Hospital of Southwest Medical University, Luzhou, Sichuan, China; ^4^ Department of Plastic Surgery, The Second Affiliated Hospital of Army Medical University, Chongqin, China

**Keywords:** superficial inferior epigastric artery (SIEA) flap, lower limb and foot, soft tissue defects, reconstruct, surgery

## Abstract

**Background:**

Large soft tissue defects of the lower limb and foot are common occurrence in clinical practice and a considerable number of flaps have been used to treat them. However, there have been few reports using the superficial inferior epigastric artery (SIEA) flap. This review aims to present the experience of using the SIEA flaps for the repair of large soft tissue defects of the lower limb and foot.

**Methods:**

A retrospective review of data from 11 patients who underwent coverage of lower limb and foot defects exceeding 120 cm^2^ (15 × 9 cm) using SIEA flaps from March 2018 to July 2022 were retrospectively reviewed. The average size of the defects was 18 × 11 cm^2^ (range 15 × 9 cm^2^–32 × 16 cm^2^). Flap survival rates, surgical complications and overall long-term outcomes were recorded.

**Results:**

All 11 flaps survived. One flap was partially necrotic at the edge and healed after several changes of dressing. Additionally, one flap presented with mild venous congestion. The mean follow-up period was 18 months (ranging from 12 to 30 months). The mean size of the flaps was 20 × 12 cm^2^ (range 17 × 9 cm^2^–34 × 18 cm^2^). The flaps were observed to be aesthetically pleasing and exhibited a well-defined texture. The donor wounds were successfully closed primarily, with only linear scarring remaining.

**Conclusions:**

The SIEA flap is characterised by concealed donor area, superficial vascular location, easy access and primary closure, which results in favourable aesthetic outcomes. It is an appropriate choice for the repair of large soft tissue defects of the lower limb and foot.

## Highlights

1. Previous reports have shown that the superficial inferior epigastric artery (SIEA) flap is limited to breast reconstruction, head and neck, oral wound repair and small soft tissue defects of the extremities, the use of the SIEA flap to cover large defects now offers a new solution to a clinical problem.
2. The SIEA flap has a concealed donor area, superficial vascular location, easy access and primary closure, Showing a better aesthetic effect.

## 1 Introduction

The reconstruction of large traumatic soft tissue defects in the limb and foot is a challenge for surgeons, frequently necessitating the use of free flaps for coverage. The traditional repair options are the anterolateral thigh flap, the latissimus dorsi flap and some combination flaps (1–3). Currently, there is a considerable market for flaps using the abdomen as the donor area due to their concealed location and low donor impact, and the SIEA flap is one such flap.

Previous reports have shown that the SIEA flap is limited to breast reconstruction, head and neck, oral wound repair and small soft tissue defects of the extremities, due to the evident advantages and disadvantages associated with its use (4–6). However, with the development of microtechnique and more profound comprehension of abdominal vascular anatomy, the SIEA flap is also a potential option for covering miscellaneous and large defects. Moreover, there is a paucity of literature documenting the utilisation of SIEA flaps to cover large lower limb and foot soft tissue defects. Based on the concept of aesthetic repair with minimal impact on the donor area, this paper presents our experience of utilising the SIEA flap to repair large soft tissue defects in the lower limb and foot.

## 2 Patients and methods

From March 2018 to July 2022, 11 patients with large soft tissue defects of the lower limbs and feet underwent SIEA flap reconstruction (defects exceeding 100 cm^2^ in size are defined as large defects), in accordance with the approval of the hospital review committee. All patients provided written informed consent to participate in the study. Medical history and statistical data for each patient, including gender, age, comorbidities, defect location, defect size, flap size, complications and follow-up data were obtained by retrospective chart review (Table 1).

**Table 1:** Patient characteristics.

Items	Value
Gender (*n*)	
Male	8
Female	3
Age (years)	43.1 (ranged, 21–69)
Etiology (*n*)	
Traumatic	6
Wound infection	3
Burn	2
Defect size (cm^2^)	18 × 11 (ranged, 15 × 9–32 × 16)
Flap size (cm^2^)	20 × 12 (ranged, 17 × 9–34 × 18)
Flap type (*n*)	
Single pedicle SIEA flap	9
Bi-pedicled SIEA flap	2
Complication (*n*)	
Distal necrosis	1
Follow-up time (month)	18 (12–30)

### 2.1 Preoperative preparations

The initial assessment of patients with large defects in the lower limb or foot entailed an evaluation of the traumatic injury and the patient's flap donor area. Patients were treated with emergency debridement at stage I, with the objective of completely removing contaminated necrotic tissue. In cases of combined fractures, the bones were realigned and secured, while ruptured tendons and vital vessels and nerves were promptly repaired. Antibiotic-loaded bone cement was used to fill the bone defect, and if the wound was heavily contaminated, continuous debridement was necessary. Vacuum sealing drainage was employed to maintain the wound's cleanliness and moisture, along with empirical broad-spectrum antibiotic treatment. Surgical intervention was conducted only when the wound was deemed satisfactory.

All patients underwent Doppler or computed tomography-assisted angiography (CTA) to ascertain the presence of SIEA in the abdominal wall and to determine its vascular diameter, course, distribution of penetrating branches and to initially mark the vascular alignment. The study included SIEA with a minimum outer diameter of 1.5 mm at the lower abdominal incision was only included.

### 2.2 Surgical technique

A pneumatic tourniquet was applied under general anaesthesia, and the surgeon delineated the defect on the abdomen and outlined the SIEA flap at the designated mark, selecting a size that was slightly larger than the area affected by trauma. The flap was selected for placement between the bi-inguinal ligaments and the umbilicus, ensuring that the upper and lower edges of the flap are situated at a distance from the navel and perineum. The femoral artery identified at a depth of 2.5 cm below the midpoint of the inguinal ligament and the SIEA flap was prepared deep within the vessel. The skin and subcutaneous tissues were incised and the dorsal side of the pedicle was maintained in adherence to the rectus fascia to reduce tension at the pedicle and reduce the incidence of vasospasm. The SIEA, the superficial inferior epigastric vein (SIEV), and the thick subcutaneous vein were identified by referencing to the location of the preoperative markings and the subcutaneous depth of the vessels that had already been explored. The pedicle of the vessel was isolated from the main femoral artery vessel. The vessel diameter and pulsation are measured again and the pedicle is protected with a small muscle cuff. The flaps were peeled along the designated line of incision, and a a small quantity of lymphatic tissue could be transported along with the graft, contingent upon the requirements of the wound. Excess adipose tissue was removed under the microscope along the vascular pedicle to thin the flap. Following the successful harvesting of the flap, the primary closure of the donor site was carried out. When flaps are transplanted to the recipient area, as the recipient area varies, the selected recipient vessel should be based on the conditions. In the cases we provided, the distal posterior tibial artery, anterior tibial artery, and posterior tibial artery were selected as the anastomotic vessels for the recipient vessels. Common protocols include but are not limited to: end-to-end anastomosis of the SIEA with the distal anterior tibial artery on one side and the distal posterior dorsal artery on the other side; end-to-end anastomosis of the superficial vein of the abdominal wall with the corresponding companion vein; end-to-end anastomosis of the subcutaneous vein with the proximal saphenous vein or saphenous vein; and so on. Venous anastomoses can be done with the anastomosis Coupler, but arterial anastomoses are recommended to be hand-sewn for safety. In two of these cases, we chose a bi-pedicled SIEA flap carrying bilateral SIEAs as a cover due to its peculiar location. Following the surgical procedure, the flap was monitored for oxygen saturation, flap appearance colour, and temperature to ensure flap survival.

## 3 Results

All flaps survived. 9 of the 11 flaps were reconstructed with single pedicle SIEA flaps and 2 with bi-pedicled SIEA flaps. One case of partial necrosis of the flap edge was healed after several dressing changes. There were no postoperative complications, including wound infection, and no further surgery was required in all patients. The mean size of the flaps was 20 × 12 cm^2^ (range 17 × 9 cm^2^–34 × 18 cm^2^). 11 patients were followed up for 12–36 months after surgery, with a mean of 18 months. The flaps were aesthetically pleasing, well textured and functionally satisfactory. The area from which the donation was taken has healed, leaving only linear scars.

## 4 Case reports

### 4.1 Case 1

**Figure 1:**
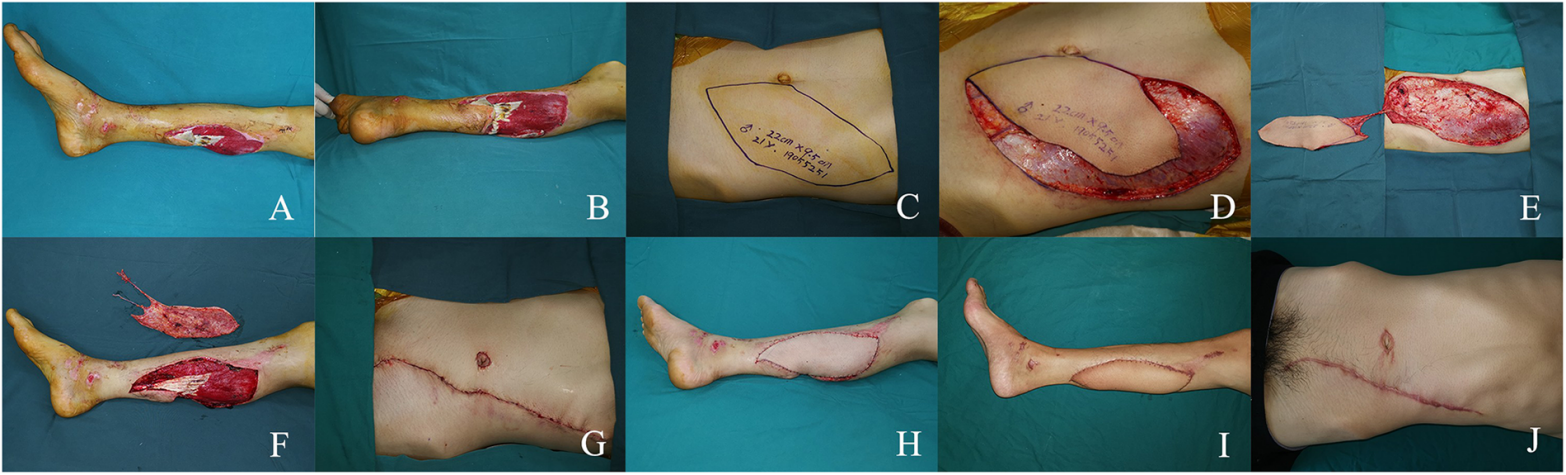
**(A,B)** large soft tissue defect in the left medial calf. **(C)** Preoperative design. **(D,E)** Intraoperative flap dissection. **(F)** Intra-flap anastomosis. **(G,H)** Immediate postoperative appearance. **(I,J)** Views at the 6-month postoperative follow-up.

A 21-year-old man suffered a traumatic injury resulting in a large soft tissue defect on the posterior aspect of his right calf. After thorough debridement, an extensive soft tissue defect with exposed tendons was left (Figures 1A,B). A 22 × 9.5 cm SIEA flap was designed on the left abdomen and transferred to cover the defect (Figures 1C–E). For flap grafting, the SIEA pedicle was anastomosed to the posterior tibial artery in an end-to-side manner (Figures 1F,G). Primary closure was performed at the donor site (Figure 1H). The flap survived successfully and 6 months after the procedure, the patient regained function and was satisfied with the appearance (Figures 1I,J).

### 4.2 Case 2

**Figure 2:**
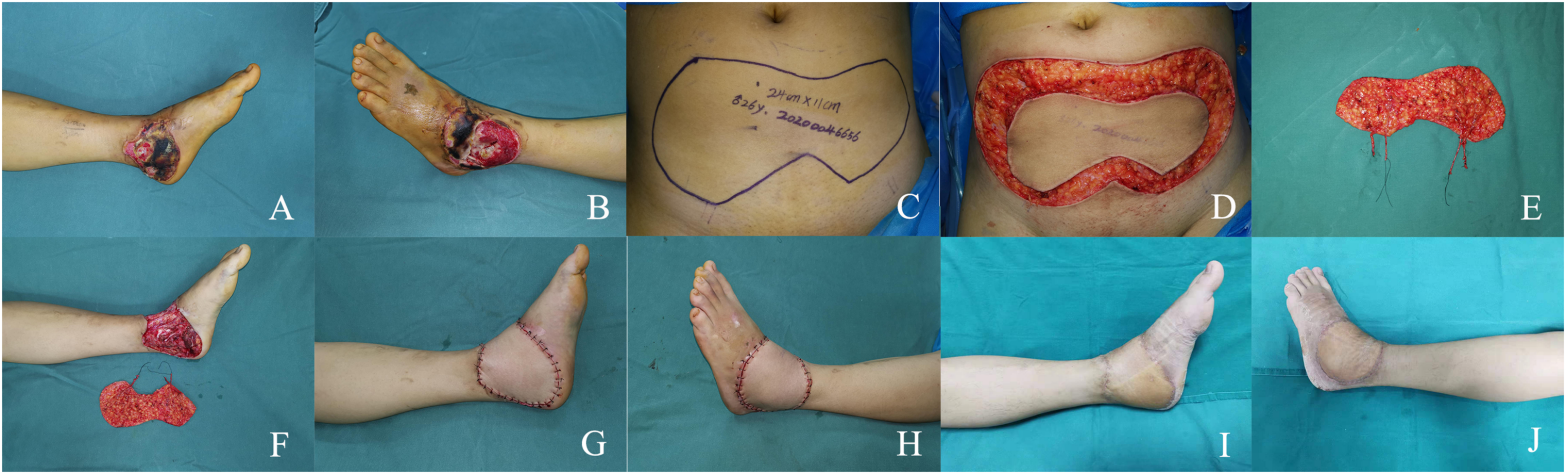
**(A,B)** large soft tissue defect on the medial and lateral aspect of the left ankle. **(C)** Preoperative design. **(D,E)** Intraoperative flap dissection. **(F)** Intra-flap anastomosis. **(G,H)** Immediate appearance of the recipient site. **(I,J)** Views at the 18-month postoperative follow-up.

A 26-year-old man suffered a burn resulting in a large soft tissue defect on the medial and lateral aspect of his left ankle (Figures 2A,B). After thorough debridement, an extensive non-contiguous soft tissue defect was left with the joint capsule and tendons exposed. A SIEA bi-pedicled conjoined flap was taken, with a flap size of 24 × 11 cm (Figures 2C–E). Before the flap was grafted, the arterial branches of the SIEA pedicles on both sides were T-connected, and then the main stem of the right SIEA pedicle was anastomosed to the posterior tibial artery in an end-to-side manner (Figures 2F–H). The flap survived successfully and 18 months after surgery, the patient regained ankle function and was satisfied with its appearance (Figures 2I,J).

## 5 Discussion

Large soft tissue defects of the lower limb and foot are frequently the consequence of high-energy trauma, chronic infection and tumours and present a significant challenge in treatment (7, 8). The selection of an appropriate flap should be based on a combination of factors, including the shape of the flap, the vascular conditions present, the implications for the donor site and the surgical difficulty involved. The choice of donor site is of particular importance, as it can significantly influence the overall outcome of the procedure. As microsurgery continues to evolve and the aesthetic requirements become more demanding, flaps using the abdomen as the donor area are gaining popularity among a growing number of practitioners.

The SIEA flap was first used in 1971 by Antia and Buch (9) for the repair of facial defects, and in 1975 Taylor and Daniel (10) first described the anatomical relationship of the SIEA, which offered the possibility of transplantation as a free flap. Due to the simplicity of excision, large volume of tissue and minimal donor damage, the SIEA flap has become a well-established technique for reconstruction of the head, face and autologous mammary gland over the past decade (11, 12). In comparison to the deep inferior epigastric perforator (DIEP) flap, which is also a donor to the lower abdomen, the SIEA flap has an ideal flap texture and a vascular tip that runs superficially through the rectus abdominis muscle, thereby avoiding intraoperative injury to the rectus abdominis muscle. This has contributed to the increasing popularity of the flap in recent years, as well as its use for various soft tissue defects in the limbs (13, 14). Miyamoto et al. (15) used the SIEA flap to transplant small defects of the limbs following resection of Soft Tissue Sarcoma, all with satisfactory results. The SIEA flap offers certain advantages in the repair of defects; however, its anatomical variability and relatively small vascular diameter restrict its application to some extent.

The SIEA has its origin in the femoral artery and forms a common trunk with the SCIA. It travels medially for a distance of between 1 and 2 cm to the midpoint of the inguinal ligament, crossing Scarpa's fascia and subsequently proceeding deep into the subcutaneous tissue. Here, its perfusion generally does not extend beyond the abdominal midline, nourishing the soft tissues of the lower abdominal skin with the DIEP. The anatomy of this flap has been the subject of extensive study and reporting in the literature. However, due to the high anatomical variability of the SIEA vessels, the exhibits considerable variation in terms of its defect rate and mean canal diameter. In 1975 Taylor and Daniel (8) observed a 35% absence rate of the SIEA in 100 human specimens dissected. Nevertheless, some recent studies have evidenced the presence of SIEA to be over 90%, with an average calibre of 0.6–2 mm (16–19). The findings suggest that SIEA is more consistently present and larger in calibre than previously reported, allowing surgeons to gain a clearer understanding of the anatomy of SIEA and suggesting that there may be further clinical applications. Despite the uncertainty of its vascular condition, the key to using this flap hinges upon the preoperative assessment of the vascularity. In this study, it was observed that 88% of patients met the requisite vascular conditioning standards, with a mean internal diameter of 1.97 ± 0.74 mm for the SIEA and 1.78 ± 0.69 mm for the SIEV. To guarantee the survival of the flap and minimise the incidence of complications, we suggest that a measurement of 1.5 mm at the level of the inguinal ligament is a watershed.

Another issue of concern to us is the area of the SIEV that can be nourished. In previous experience, a single vessel did not act as a pedicle for all the soft tissue on one side of the lower abdomen. The principle of excision of the lower abdominal flap is mainly based on the theory of blood supply zoning in the lower abdomen, and the area nourished by the SIEV is not a dominant area (20, 21). In our study, the use of SIEA as the sole blood supply allowed nutrition of flaps over an area of 120 mm. And the SIEA is richly anastomosed with the DIEP, the SICA and the posterior intercostal artery in the fascial layer, and the flap can be designed to overlap with the SIEA flap to facilitate intraoperative conversion to an alternate plan for flap removal, when the flap needs external pressure it can carry the artery here.

In our study, the overall complication rate of the SIEA flap remained low, with only one postoperative case of partial flap loss observed. No wound infections, haematomas, fat necrosis or hernias/bulges were observed in the group. In previous reports of reconstructions using SIEA flaps, thrombosis has been identified as the most common complication, leading to possible total flap loss. Sarik et al. (22) used 47 SIEA flaps for breast reconstruction, with an intraoperative thrombosis incidence of 17.0% and a total flap loss rate of 2.9%. They concluded that these incidences remain higher than other abdominal-based autologous breast reconstruction techniques and do not appear to be attributable to an operator learning curve. Furthermore, Some investigators have proposed that complications and failures of the SIEA flap can be attributed to arterial thrombosis (13). We consider that the higher rate of vascular thrombosis may be related to several factors, including high vascular variability, inadequate preoperative assessment, and limited surgeon experience.

It is equally important to consider the empirical details of the use of SIEA flaps in trauma reconstruction in order to reduce the incidence of flap failure. As the SIEA crosses the inguinal ligament and travels further towards the epidermis, the calibre of the vessels becomes finer. It is crucial to ensure that the subcutaneous incision is positioned as far down as possible and that the separation is made close to the origin of the vessel pedicle in order to maximise the length and calibre. Following the subcutaneous freeing of the vascular pedicle, the flap near the tip can be de-epithelialized and buried in the tissue to act as a pedicle length. If the SIEA and SCIA share a common trunk, it may not be necessary to ligate it intraoperatively. An alternative approach is to modify the flap slightly laterally, utilising the superficial iliac vein in a adjunctive manner with the flap return vein to enhance flap survival. The clinical selection of the SIEA flap necessitates a degree of flexibility in design and excision, taking into account the potential for anatomical variation based on the findings of the preoperative examination and intraoperative observations.

Flaps used to cover lower limb trauma can be considered to carry a small amount of inguinal lymphatic tissue, which can effectively increase lymphatic return within the flap and reduce postoperative swelling in the affected area of the lower limb. The SIEA travels superficially in the abdominal fat and can also be thinned in obese abdominal patients depending on the trauma required, and after thinning of the flap, careful haemostasis is required to avoid subcutaneous haematoma.

## 6 Conclusions

The SIEA flap may represent a viable option for reconstructing large soft tissue defects in the lower limbs and foot. The advantages of this procedure include the simplicity of excision, limited damage to the donor area and the possibility of excising a relatively large area. Nevertheless, the vascular condition of the SIEA needs to be screened prior to surgery and care should be taken at each step of the procedure to improve the survival rate of the flap. This study is limited by its retrospective analysis of cases, lack of a control group, and small sample size.

## Data Availability

The raw data supporting the conclusions of this article will be made available by the authors, without undue reservation.
